# Insulin and the Lung: Connecting Asthma and Metabolic Syndrome

**DOI:** 10.1155/2013/627384

**Published:** 2013-09-24

**Authors:** Suchita Singh, Y. S. Prakash, Allan Linneberg, Anurag Agrawal

**Affiliations:** ^1^Center of Excellence for Translational Research in Asthma & Lung Disease, CSIR-Institute of Genomics and Integrative Biology, New Delhi 110007, India; ^2^Department of Anesthesiology, Mayo Clinic, Rochester, MN 55905, USA; ^3^Department of Physiology & Biomedical Engineering, Mayo Clinic, Rochester, MN 55905, USA; ^4^Research Center for Prevention and Health, Glostrup University Hospital, Glostrup 2600, Denmark

## Abstract

Obesity, metabolic syndrome, and asthma are all rapidly increasing globally. Substantial emerging evidence suggests that these three conditions are epidemiologically and mechanistically linked. Since the link between obesity and asthma appears to extend beyond mechanical pulmonary disadvantage, molecular understanding is necessary. Insulin resistance is a strong, independent risk factor for asthma development, but it is unknown whether a direct effect of insulin on the lung is involved. This review summarizes current knowledge regarding the effect of insulin on cellular components of the lung and highlights the molecular consequences of insulin-related metabolic signaling cascades that could adversely affect lung structure and function. Examples include airway smooth muscle proliferation and contractility and regulatory signaling networks that are associated with asthma. These aspects of insulin signaling provide mechanistic insight into the clinical evidence for the links between obesity, metabolic syndrome, and airway diseases, setting the stage for novel therapeutic avenues targeting these conditions.

## 1. Introduction

It is now well recognized that obesity and asthma are epidemiologically linked [1–4]. Such a relationship is also seen between asthma and other markers of the metabolic syndrome such as insulin resistance and hypertension that cannot be accounted for by increased body mass alone [4–7]. While both obesity and asthma are individually associated with an increased state of inflammation [8], interestingly, in obese asthmatics, there is a dissociation between cellular inflammation and severity of symptoms, especially in women [9, 10]. This discordance would suggest that while obesity-related systemic inflammation can certainly be one mechanism for increased asthma risk, there is a need to examine mechanisms independent of cellular inflammation that may play a role in asthma in the context of conditions such as obesity and metabolic syndrome.

A number of cellular signaling and metabolism mechanisms could contribute to increased asthma risk in patients with obesity and/or metabolic syndrome. Considering the fact that altered glucose metabolism occurs in both cases, and hyperinsulinemia with reduced insulin sensitivity is involved, an obvious potential factor affecting the lung is insulin itself, particularly a direct effect on structural cells as well as immune cells in the airway. In a large Danish cohort, it was observed that insulin resistance (IR) was more strongly related to asthma risk than any of the anthropometric parameters [11]. While this study did not specifically examine serum insulin, independent of blood glucose or diabetes, it is recognized that insulin resistance (IR) and consequent hyperinsulinemia are central molecular pathologies in the genesis of the metabolic syndrome [12, 13]. Other markers of metabolic syndrome such as C-reactive protein and correlates such as hyperglycemia, diabetes, or hypertension have all been associated with reduced lung function, asthma [14], or even COPD [15], in large clinical studies. Yet the direct impact of hyperinsulinemia and IR on lung function is poorly understood. If insulin excess can directly alter lung cellular physiology, this would represent a fundamental common molecular link between asthma and the cardiometabolic syndrome [16].

This review focuses on the current stage of knowledge regarding the direct effects of insulin in lung cells in the context of airway remodeling and hyperresponsiveness. Here, it is important to emphasize that in fact there is a significant knowledge gap regarding insulin effects in the airway, and we therefore draw upon what is known in other cell types to generate hypotheses that could drive future research. Certainly, our focus on insulin does not rule out several other potential mechanisms such as dysfunctional arginine metabolism and uncoupling of nitric-oxide synthase (NOS) by increased asymmetric dimethyl arginine (ADMA) [17], effects of adipokines, and direct mechanical effects of thoracoabdominal obesity on lung mechanics. These important topics are reviewed in detail elsewhere in this issue. 

## 2. Insulin and IR

Insulin is one of the central homeostatic hormones with global effects that extend beyond glucose and lipid metabolism. As a pleiotropic hormone [18], insulin effects range from the well-known hypoglycemia to regulation of cell growth and differentiation [19, 20]. Insulin regulates a number of key metabolic biological processes such as stimulation of glucose uptake, lipid synthesis, oxidation, storage of fat, and cell proliferation [21–23]. Insulin-mediated signaling varies significantly between cells and tissues necessitating an understanding of its actions in the context of the lung and in specific cell types within the lung. Insulin resistance (IR), that is, reduced responsiveness to insulin in liver, muscle, and adipose tissue, is closely associated with various metabolic diseases such as obesity, metabolic syndrome, nonalcoholic fatty liver disease [24], and type 2 diabetes mellitus [25]. Since IR in key metabolic tissues is associated initially with compensatory hyperinsulinemia, insulin-related effects that retain sensitivity in other tissues are expected to be increased, even in the face of metabolic IR. This is a partial reflection of distinct physiological processes in multiple organs [26] and is important because IR is also associated with other putatively nonmetabolic diseases such as asthma [4, 27] and some cancers [28–32]. While relative deficiency of insulin and hyperglycemia are well studied in diabetes, the harmful effects of insulin excess are poorly recognized except where they result in hypoglycemia. Importantly, given the cell- and tissue-specific heterogeneity in insulin signaling and the potential confounding role of disease states *per se*, effects on other tissues may not be easily extensible to the lung. Nonetheless, considerable insight into potential mechanisms by which insulin influences lung cellular components relevant to asthma may be gleaned from such prior data. 

## 3. Insulin and the Lung

Expression of insulin receptors in the lung has been verified [33]; however, their role has only been partially characterized using crude membrane of normal lung as well as plasma membrane fractions of lung tissue. Importantly, interaction of these receptors with insulin appears to be time and temperature dependent, and furthermore rapid, saturable, and highly reversible [34, 35]. The relevance of these findings is that insulin has the potential to dynamically influence lung structure and function at various life stages and thus modulating asthma predisposition.

Insulin receptors are important during lung development with lung epithelial cells abundantly expressing insulin receptors during the pseudoglandular stage with receptor levels decreasing during later stages of development [36]. Assuming that maternal insulin is the agonist, these data fit well with prior evidence that maternal diabetes has substantial growth effects on fetal lungs. Miakotina et al. and others have shown that high insulin levels delay lung development in fetuses of diabetic mothers by inhibiting surfactant protein A (SP-A) [37]. Inhibition of SP-A and SP-B genes' expression leads to increased incidence of respiratory distress syndrome (RDS) in infants of diabetic mothers [38]. In a large Canadian study, asthmatic children were more likely to be of diabetic mothers than children without asthma [39]. What is less clear, but is likely, is whether these diabetic mothers had hyperinsulinemia [40]. Furthermore, alternative mechanisms may be at play, including hyperglycemia, and altered cytokine/adipokine milieu. 

While these clinical associations are clear, there is limited experimental evidence supporting a direct role for insulin *per se* in lung development. In cultured lung epithelial cells, insulin reduces VEGF expression and transcriptional activity of HIF-2 on *VEGF* promoter in an mTOR-dependent manner. The importance of the Akt-mTOR pathway in lung epithelium relates to the pathogenesis of infant RDS [41] which predisposes towards asthma later in life. Animal models of hyperinsulinemia are more complex with at least one report of accelerated fetal lung maturation in pregnant rabbits by a two-day intravenous (IV) infusion of insulin, but with significant hypoglycemia [42]. Accordingly, it is difficult to conclude whether insulin is promaturation when it comes to lung development, and here it is important to consider whether the timing of high versus low level of insulin receptor expression and activation is a confounding factor. 

Other than effects of insulin on developing lungs, recent efforts towards developing inhaled insulin formulations for diabetes management have provided interesting insights into direct effects of insulin on the mature lung. Since the pulmonary epithelium and the surfactant that lines the alveoli (0.1-0.2 *μ*m thick) constitute physical barriers to pulmonary absorption, local deposition and action of insulin are to be expected. Locally high concentrations of protease inhibitors and acidic formulations seem to protect the insulin peptide from membrane-associated cells and intracellular proteases [43–45] resulting in much of the inhaled insulin being absorbed systemically in the alveolar region. However, despite good systemic delivery, there also appears to be substantial local effect of inhaled insulin. For example, inhaled insulin in diabetic patients is associated with a decrease in forced expiratory volume in 1 second (FEV1) [46, 47], but the mechanisms are not clear. Certainly, insulin can shift T cells towards a Th2-type response, known to be a key event in the pathogenesis of asthma [48]. It has also been observed that insulin, via activation of PI3K pathway, promotes mast cell survival and degranulation, which facilitate bronchoconstriction [49]. Nonspecific proinflammatory effects via activation of pulmonary macrophages are also possible, and in some studies it has been shown that inhaled insulin may deposit at air-tissue interfaces with characteristics of amyloid aggregates [50]. The relevance of these findings may be in that inhaled formulations of insulin that were once promising, approved strategies for treatment of diabetes mellitus in US and Europe [51, 52] have been withdrawn due to persistent reports of respiratory problems, including cough. On the other hand, it appears that insulin also has anti-inflammatory effect in the context of severe Th1-type inflammation. Insulin has been found to reduce levels of inflammatory cytokines, attenuate acute lung injury and systemic inflammatory response, and promote survival in rodents exposed to LPS [53]. 

Collectively, these limited data again suggest a dichotomous role for insulin where increased levels of insulin in the mature (adult) lung have found to be detrimental on the one hand but protective on the other. What is important to determine is whether systemic hyperinsulinemia as occurring in metabolic syndrome leads to pathophysiological changes leading to asthma, or is protective, and the mechanisms by which insulin acts on the airway. 

## 4. Insulin and Airway Smooth Muscle

While asthma is usually defined as an inflammatory disease, a cardinal feature is airway hyperresponsiveness (AHR): excessive narrowing of airways in response to normal constrictive stimuli [54]. AHR is associated with increase in airway smooth muscle (ASM) mass, dysfunction of bronchial epithelium, and alterations in extracellular matrix (ECM) within the airway wall. Increased ASM mass is considered to play a key role in the development of AHR [55] and activate epithelial mesenchymal trophic unit (EMTU) that leads to airway remodeling.

As a growth factor, the contribution of insulin to increased ASM mass and/or contractility in the context of asthma is obviously important.

**Figure 1:**
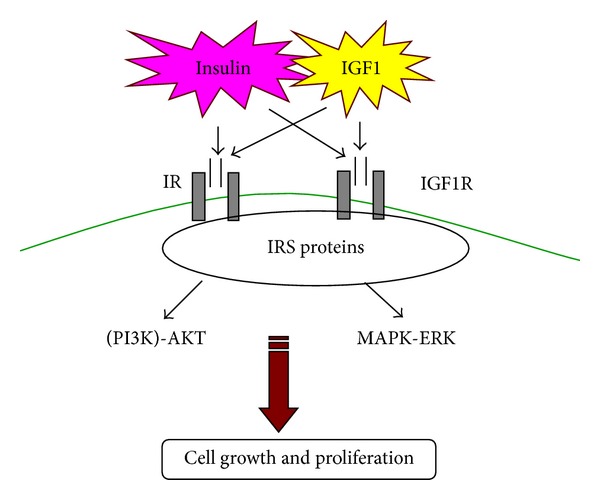
Insulin and IGF-1 can crossactivate each other's receptors, that is, insulin-IGF1R, IGF1-IR, which can lead to insulin-mediated cell growth and proliferation.

Noveral and colleagues first showed that functional insulin-like growth factor-1 (IGF-1) receptors are present on rabbit ASM and that their stimulation is sufficient to induce ASM proliferation [56, 57]. These studies have been replicated in other animal model systems, and the pathway was determined to be activation of the MAP kinase system [58]. It is well known that insulin and IGF-1 can crossactivate each other's receptors (insulin receptor (InsR) and IGF1R) and that there is also significant crosstalk downstream to these receptors via the insulin receptor substrate proteins (IRS; Figure 1). It has been subsequently shown that high levels of insulin promote ASM contraction [59] and enhance contractile responses to methacholine and KCl [60, 61]. These effects have been reported to occur via Rho kinase- and PI3 kinase-dependent signaling pathways [62, 63]. Other reports suggest that insulin increases ECM proteins such as laminin (a2, b1, and g1 chain expression) [64] which are important in lung growth and differentiation of naïve mesenchymal cells, leading to hypercontractile and hypoproliferative ASM [65, 66]. Furthermore, limited studies suggest that aerosol administration of insulin leads to ASM contraction but indirectly via production of procontractile prostaglandins that involve Rho kinase [67]. Overall, the data so far suggests that, if anything, insulin effects on ASM are likely to result in increased airway contractility, cell proliferation, and fibrosis, all of which should lead to a thicker, stiffer, and hypercontractile airway reflective of an asthma phenotype. However, it is also important to recognize that much of the work has been in vitro, with high levels of insulin, typically applied for relatively short periods. Whether prolonged hyperinsulinemia and/or activation of insulin receptors leads to different cellular effects in the airway, and whether such effects are reversible (in the context of therapy) remain to be determined. 

## 5. Insulin and PI3/Akt Signaling

PI3K/Akt signaling has a central role in the conserved downstream pathway of insulin signaling [68, 69] and is an important regulator of diverse array of cellular events, including cell growth and cell survival in a number of cell types [70]. Several studies have validated the functional significance of the PI3K pathway in glucose homeostasis and shown that PI3K inhibition leads to insulin resistance [71, 72]. 

It has been also shown that insulin is a potent activator of PI3K in human bronchial epithelial cells and inhibits TLR3 mediated apoptosis [73]. It has been also shown that insulin, via activation of PI3K pathway, promotes mast cell survival and degranulation, which may leads to bronchoconstriction [49].

**Figure 2:**
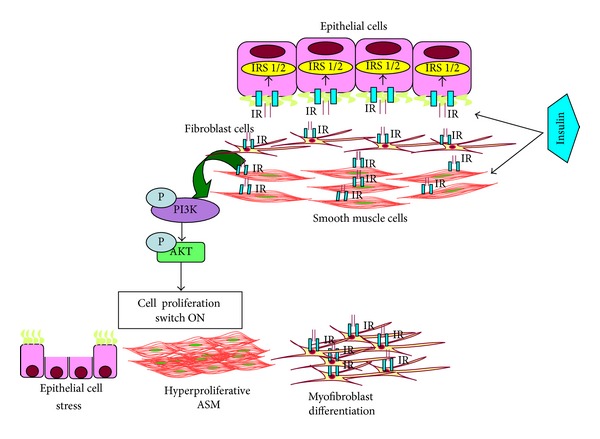
A schematic model of how insulin may be involved in regulation of asthma-like changes in lungs.

 This pathway is also thought to be important at least in prenatal lung development in the context of maternal diabetes where fetal hyperinsulinemia in response to maternal hyperglycemia [74–77] results in PI3K/Akt1/mTOR activation and induces RDS [78]. Also, as mentioned previously, high levels of insulin through the PI 3-kinase signaling pathway may also inhibit surfactant protein production expressed in the lung epithelial cells and lung maturation [37, 79, 80], thus predisposing the immature lung to airway diseases later in life. While these data relate to the developing lung, the relevance of the enhanced PI3/Akt signaling also lies in its well-recognized role in adult asthma. For example, loss of function mutations in the principal inhibitory phosphatases SHIP, PTEN, and INPP4A, are associated with asthma [81], and knockdown of INPP4A induces airway remodeling and hyperresponsiveness. Activation of the PI3/Akt pathway promotes survival of airway epithelial cells as well as ASM and conversely can enhance proliferation [82], thus contributing to airway remodeling. Whether insulin activation of PI3K/Akt is involved in this regard is not currently known. However, insulin has been shown to act via PI3/Akt to inhibit epithelial apoptosis that normally occurs with viral exposure and could thus promote airway remodeling in this context (Figure 2).

## 6. Insulin, Wnt/*β*-Catenin Signaling, and Airway Remodeling

Some studies have also reported insulin regulation of Wnt/*β*-catenin pathway and stimulation of transcription of Lef/Tcf-dependent genes via activation of PI3K/Akt and Ras signaling pathways which normally inhibit GSK3-*β* and activate the *β*-catenin pathway in hepatocarcinogenesis [83]. Dysregulation of Wnt/*β*-catenin pathway influences many biological processes, including cell fate decisions, stem cell proliferation [84], and axis specification [85–88]. While relationships between insulin and Wnt/*β*-catenin have not been reported in the lung *per se*, Wnt/*β*-catenin pathways are increasingly recognized as being important in regulation of lung cell proliferation and differentiation [89]. *β*-catenin is required for normal lung morphogenesis, and deletion of *β*-catenin during critical periods of lung development leads to blocked alveolar epithelial cell differentiation and disruption of alveolar formation [87]. Furthermore, conditional activation of *β*-catenin in respiratory epithelial cells leads to altered epithelial cell differentiation by induction of alveolar marker prosurfactant protein C (SP-C) and causes goblet cell hyperplasia, air space enlargement, and pulmonary tumors. Thus, *β*-catenin signaling pathway has a critical role in the differentiation of the respiratory epithelium in the postnatal lung [90]. Accordingly, even during lung development, insulin could potentially promote morphogenesis via activation of the Wnt/*β*-catenin pathway, although it would be important to determine whether the relative timings of insulin versus Wnt/*β*-catenin activation are detrimental or beneficial. 

In the adult airway, activated *β*-catenin-/TCF-/LEF-dependent gene transcription of VEGF [91], matrix proteins such as fibronectin and versican, and proinflammatory enzymes/mediators such as cyclooxygenase (COX)-2 [92] and interleukin (IL-8) suggest the involvement of this pathway in regulation of inflammation and airway remodeling. Here too, insulin-mediated activation of *β*-catenin signaling could be involved in airway disease pathogenesis. While there is currently no information in the context of asthma *per se*, data from fibroproliferative lung diseases may be suggestive where activation of *β*-catenin signaling is involved [93]. Inhibition of GSK3*β* and stabilization of *β*-catenin is governed by TGF-*β* signaling and leads to altered ECM [94]. Of relevance, diabetes and metabolic syndrome have been associated with increased risk of idiopathic pulmonary fibrosis and COPD [95, 96]. Whether insulin *per se* plays a role in these diseases is not known, but if so, modulation of Wnt signaling pathway may be relevant.

Also, in other systems like the heart, Akt-mediated GSK3*β* phosphorylation leads to increased *β*-catenin expression and causes cardiac smooth muscle hypertrophy [97], whether this may be mirrored in the airway smooth muscle, remains to be determined.

## 7. Conclusion

There is substantial data that mechanistically links insulin and insulin like growth factor-1 to lung development and function. It is conceivable but not proven that hyperinsulinemia may lead to development of lung disease, particularly asthma. Experimental studies that directly address this possibility are much needed.
